# A Multivariate Analysis of “Metabolic Phenotype” Patterns in Children and Adolescents with Obesity for the Early Stratification of Patients at Risk of Metabolic Syndrome

**DOI:** 10.3390/jcm11071856

**Published:** 2022-03-27

**Authors:** Valeria Calcaterra, Giacomo Biganzoli, Simona Ferraro, Elvira Verduci, Virginia Rossi, Sara Vizzuso, Alessandra Bosetti, Barbara Borsani, Elia Biganzoli, Gianvincenzo Zuccotti

**Affiliations:** 1 Pediatric and Adolescent Unit, Department of Internal Medicine, University of Pavia, 27100 Pavia, Italy; 2 Pediatric Department, “V. Buzzi” Children’s Hospital, 20154 Milano, Italy; elvira.verduci@unimi.it (E.V.); virginia.rossi@unimi.it (V.R.); sara.vizzuso@asst-fbf-sacco.it (S.V.); alessandra.bosetti@asst-fbf-sacco.it (A.B.); gianvincenzo.zuccotti@unimi.it (G.Z.); 3 Department of Clinical Sciences and Community Health & DSRC, University of Milano, 20122 Milano, Italy; giaco.biganzoli@gmail.com (G.B.); elia.biganzoli@unimi.it (E.B.); 4 Department of Laboratory Medicine in Endocrinology Laboratory Unit, “Luigi Sacco” University Hospital, 20157 Milan, Italy; simona.ferraro@asst-fbf-sacco.it; 5 Department of Health Sciences, University of Milano, 20146 Milano, Italy; 6 Department of Biomedical and Clinical Science “L. Sacco”, University of Milano, 20157 Milano, Italy

**Keywords:** obesity, pediatrics, metabolic syndrome, metabolic phenotype, children

## Abstract

Background: Metabolic syndrome (MS) is closely linked to obesity; however, not all individuals with obesity will develop obesity-related complications and a metabolically healthy obesity (MHO) group is also described. Objective: To perform a multivariate analysis (MVA) of the anthropometric and biochemical data in pediatric patients with obesity to reveal a “phenotype” predictive for MS. Methods: We analyzed 528 children with obesity (OB) and 119 normal-weight pediatric patients (NW). Adiposity indices were recorded, and MS was detected. MVA was performed. Results: Analysis of the structure of correlation of the variables showed that the variables of waist circumference (WC), body mass index (BMI), and estimated fat mass (eFM) were positively correlated with each other as a whole. In addition, the variables of the triglycerides (TG), triglyceride–glucose (TyG) index, and visceral adiposity index were positively correlated with each other as a whole, although none were correlated with the variables of BMI z-score, waist-to-height ratio, WC, eFM, or weight. The variables that related to insulin resistance (IR) and dyslipidemia were crucial for the early stratification of patients at risk of MS. Conclusions: Independently of body weight, IR, dyslipidemia, hypertriglyceridemia, and fat distribution seem to be the strongest MS risk factors. The early detection of and intervention in these modifiable risk factors are useful to protect children’s health.

## 1. Introduction

Childhood obesity is one of the most serious public health issues in pediatrics [1,2,3]. The problem is global, and it is increasingly affecting many low- and middle-income countries, especially in urban settings. According to the World Health Organization (WHO), overweight and obesity are defined as abnormal or excessive fat accumulation that presents a risk to health [2]. Obesity-related complications can be identified at an early age, with important health and economic consequences [4].

In particular, metabolic syndrome (MS) has been closely linked to overweight and obesity in pediatrics. The prevalence of MS increases with the severity of obesity [5], and is a predictor of type 2diabetes (T2D), cardiovascular diseases (CVDs), and all-cause mortality in adults [4]. MS prevalence in pediatrics ranges from 0.3% to 26.4%, according to the large number of different pediatric definitions of MS and several population studies [6]. Dysglycemia/insulin resistance (IR), hypertension, high levels of triglycerides (TG), and low high-density lipoprotein cholesterol (HDL cholesterol) are included as inter-related components of MS [7,8,9,10,11,12,13,14].

The pathogenesis of MS is complex, and many aspects have still not fully been elucidated [14,15]; body composition and body-fat distribution are critical actors in the risk of development of insulin resistance (IR) [16]. Body mass index (BMI), waist circumference (WC), and waist to height ratio (WHtR) are usually considered good markers of MS [17]. Novel adiposity indices, including body shape index (ABSI), triponderal mass index (TMI), conicity index (ConI), Visceral Adiposity Index (VAI), and non-linear equations to estimate fat mass (eFM), have recently been proposed as markers of cardiometabolic risk [17,18,19,20,21,22,23,24,25,26].

However, not all individuals with obesity will develop obesity-related complications. According to the literature, a metabolically healthy obesity (MHO) group can be described [27]. The underlying mechanisms of metabolic regulation are unclear; adipokine levels, visceral fat contents, percentages of ectopic fat, and different biochemical profiles have been reported in MHO individuals compared with patients with metabolic complications [28]. The definition of clinical patterns related to metabolic risk in pediatrics may lead to more appropriate health preventive strategies.

Multivariate analysis (MVA) is used in medical research due to its ability to explore the structure of correlations and retrieve relevant associations within the data [29,30]. Studies on the inter-relationship of new adiposity indices with different metabolic phenotypes and cardiometabolic risk markers, according to sex, are limited in pediatrics [31].

We performed an MVA of the anthropometric and biochemical parameters, i.e., the “metabolic phenotype”, in children and adolescents with obesity, in order to identify clinical patterns among highly interrelated clinical variables and biomarkers predictive of MS. The early detection of specific metabolic phenotypes may be important for the tailored treatment and care of pediatric patients with obesity.

## 2. Materials and Methods

### 2.1. Patients

We retrospectively analyzed 528 Caucasian children and adolescents (274 females and 258 males, aged 11 years old (interquartile range (IQR) 9–12.2 years) with obesity referred to the Vittore Buzzi Children’s Hospital, Milan and the San Paolo University Hospital of Milan, by their general practitioner or primary care pediatrician. Patients were referred between May 2019 and May 2021. Exclusion criteria were known secondary obesity conditions, the use of any ongoing medical therapy, and concomitant chronic or acute illnesses.

As a control group, we considered 119 healthy Caucasian children and adolescents comparable for age and sex: 59 males, 60 females, with a mean age 11 years (9–13 IQR), who were enrolled as controls for other metabolic studies. All the parents or guardians gave their consent to retrospectively enroll the subjects in other studies for clinical research purposes, epidemiology, studies of pathologies, and training, aiming to improve knowledge, care, and prevention.

In all subjects, clinical evaluations and biochemical profiles were considered.

All participants or their responsible guardians provided written consent after being informed about the nature of the study. The study (protocol numbers 2015/ST/135 MI, 2020/ST/234 MI) was approved by the institutional ethics committee, and the study was conducted in accordance with the Helsinki Declaration of 1975, as revised in 2008.

### 2.2. Clinical Examination

In all of the participants, their height, weight, pubertal stage, waist circumference (WC), WHtR, measurement, DBP and SBP were considered. Standing height was measured using a Harpenden Stadiometer, with the child in an upright position, without shoes, with their heels together and toes apart, their hands by their sides, and the head aligned in the Frankfort horizontal plane [32,33]. Weight was quantified with participants not wearing shoes and in light clothing, standing upright in the center of the scale platform [32,33]. Using a flexible inch tape, the waist circumference was measured at the midpoint between the lower border of the rib cage and the iliac crest [32,33]. Blood pressure was measured twice, consecutively, using a mercury sphygmomanometer, with an appropriately sized cuff on the right arm, which was slightly flexed at heart level. The second BP measurement was used for analysis [32,33].

Pubertal stages were classified according to Marshall and Tanner [34,35] as follows: Prepubertal stage 1 = Tanner 1; Middle puberty stage 2 = Tanner 2–3; Late puberty stage 3 = Tanner 4–5. 

BMI was calculated as the body weight (kilograms) divided by height (meters squared) and was transformed into BMI z-scores using WHO reference values [36]. 

According to the BMI z-score WHO classification [36] all subjects were classified into children with normal-weight (NW; −2 ≤ BMI z-score ≤1) and with obesity (OB; BMI z-score ≥ 2).

Adiposity indices, including ABSI; TMI; VAI; ConI; and eFM, were considered and calculated as follows:

- ABSI = 1000 × WC × Wt ^−2/3^ × Ht^5/6^ [37];
- TMI = weight (kg)/height (m)^3^ [26];
- ConI = WC/(0.109 × (Wt/Ht)^0.5^) [38];
- VAI [39]
  Male = [WC/(39.68 + (1.88 × BMI))] × (TG/1.03) × (1.31/HDL-C);
  Female = [WC/(36.58 + (1.89 × BMI))] × (TG/0.81) × (1.52/HDL-C);
- Fat Mass (eFM) = weight − exp(0.3073 × height^2^ −10.0155 × d-growth-standards/standards/body-mass-index-for-age-bmi-for-age weight − 1 + 0.004571 × weight − 0.9180 × ln(age) + 0.6488 × age^0.5^ + 0.04723 × male + 2.8055) [40] (exp = exponential function, ln = natural logarithmic transformation, male = 1, female = 0).

### 2.3. Biochemical Evaluation

At enrollment, subjects underwent a blood draw in a fasting state between 8:30 a.m. and 9:00 a.m., and the plasma glucose; insulin; triglycerides (TG); and total and HDL cholesterol levels were evaluated. As a surrogate of insulin resistance (IR), the homeostasis model assessment—insulin resistance (HOMA-IR) index and triglyceride—glucose (TyG) index were assessed as follows:

- HOMA-IR= (fasting plasma insulin (mU/L) × fasting plasma glucose (mg/dL))/405 [41];
- TyG-index= ln(fasting triglycerides (mg/dL) × fasting plasma glucose (mg/dL)/2) [41].

Metabolic syndrome was defined as the presence of at least three of the following risk factors: BMI ≥ 2 z score and/or WHtR > 0.5 [42], SBP ≥ 130 mmHg and/or DBP ≥ 85 mmHg; glycemia ≥ 100 mg/dL and/or HOMA-IR ≥ 2.5 if prepubertal stage 1 or ≥4 if pubertal stage 2, 3 [43]; HDL cholesterol <40 mg/dL in females and <50 mg/dL in males; triglycerides ≥ 100 mg/dL (<10 years) or ≥130 mg/dL (≥10 years).

In addition to BMI, we also considered WHtR, because it is helpful in detecting children with a higher likelihood of presenting metabolic and cardiovascular risks [42], and it is more appropriate than WC alone to track changes in abdominal adiposity among adolescents [44].

As previously reported [31], a pathological level of fasting blood glucose (FBG) and/or IR was used as a marker of gluco-metabolic derangement, because impaired fasting glucose is rare in childhood and IR precedes glucose abnormalities [45]. The euglycemic–hyperinsulinemic clamp is the gold standard for measuring IR; however, this method is invasive, time-consuming, and is difficult to apply with pediatric patients.

### 2.4. Statistical Analysis

Univariate statistical analyses were performed to study the anthropometric and metabolic characteristics of children with NW and OB included in the analysis. We estimated the density function of the analyzed variables by applying a nonparametric method of kernel density estimation, representing the function by means of violin graphs, including a bar graph showing the mean and standard deviation values. Thus, we obtained a complete distribution view. The graphs were grouped according to obesity status (NW, OB).

To investigate the potential association between anthropometric and metabolic variables characterizing the children, principal components’ analysis was performed on the group of the children with obesity. This method allowed us to reduce the dimensionality of the dataset by projecting each data point onto the first principal components (up to three), preserving as much variation in the data as possible. We explored the metabolic and anthropometric profiles of normal weight observations by passively projecting them onto the spaces defined by the three first PCs.

To explore possible differences and similarities among observations of OB children that might define more characterizing but hidden subgroups, a cluster analysis technique was applied to all observations in the dataset, considering all anthropometric and metabolic variables analyzed in previous analyses, for construction of the distance matrix. First, hierarchical cluster analysis using Ward’s method produced a dendrogram for estimating the number of likely clusters within the sample. We made cuts at the points of change between successive fusion levels to define likely cluster boundaries. The derived number of clusters was sub-specified using k-means cluster analysis, the main clustering technique. To achieve repeatability and stability in each model, the k-means algorithm was run 50 times with random starting points. Clusters defined by the k-means algorithm were subsequently projected into subspaces defined by principal component 1 (PC1) 1, PC2, and PC3, calculated by performing PCA only on OB children.

Further univariate statistical analysis was performed to study the different characteristics of the clusters defined in the analysis. We produced a table in which all anthropometric and metabolic variables are summarized as the mean (sd) and stratified by cluster. In addition, violin plots were produced to visually capture the differences between clusters. All the statistical analyses were performed using R software (version 4.1.2). The packages used for multivariate analysis were ‘FactoMineR’ (version 2.4), ‘factoextra’ (version 1.0.7), and ‘stats’ (version 3.6.2). 

## 3. Results

### 3.1. Univariate Analysis

For the OB group, 528 observations were available, whereas for the NW children group, 119 observations were available. The main statistics of the variables considered in the univariate analysis for both groups of children are presented in Table 1. OB and NW were comparable for age and sex. As expected, significant differences were noted for all the anthropometric and metabolic variables, between the two groups.

### 3.2. Multivariate Analysis

#### 3.2.1. Principal Component Analysis

**Figure 1:**
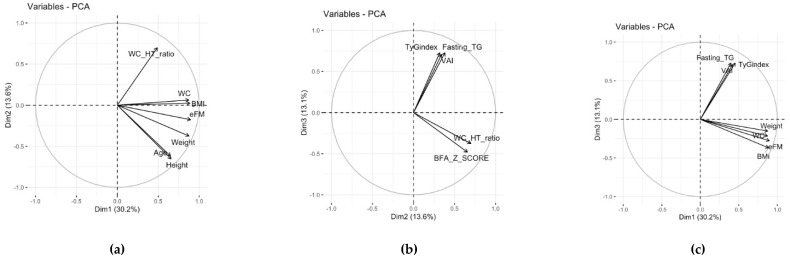
In these panels, the correlations are represented as vectors of each variable projected in the new subspaces defined by the principal components derived by the multivariate analysis (PCA). For clarity reasons, only the variables that contributed the most to the definition of the PCs and are represented more in the plane are displayed (**a**–**c**). The more the variables are correlated, the more they are projected on the same line. If they are positively correlated, they show the same direction; otherwise, they go in the opposite direction. On the other hand, if the variables are uncorrelated, they tend to project perpendicularly to form an angle of 90°.

The first three principal components explained 56% of the variance in the data (PC1 = 30%, PC2 = 13%, PC3 = 13%). The projections of the original variables onto the three planes are shown in Figure 1. The first axis (PC1) was characterized primarily by variables such as WC, eFM, BMI, and weight (which were highly correlated according to the BMI formula), but also influenced to a lesser degree by the variables of insulin and HOMA-IR (also highly correlated according to the HOMA-IR formula). The second axis (PC2) was mainly characterized by the variables WC/Ht, BMI z-score, height, and age. The third axis (PC3) was characterized to a great degree by the variables of fasting TG and TyG index, and to a smaller degree by the variable VAI.

It appeared that variables such as SBP and DBP were not well represented and did not contribute to the definition of the first three principal components.

In all the planes defined by the PCs, it appeared that the variables WC, BMI, and eFM were clustered and positively correlated with PC1. In the plane defined by PC1 and PC2 and the plane defined by PC1 and PC3, the variables of fasting TG, TyG index, and VAI were positively correlated with each other and with PC3, although neither were correlated with the variables of BMI z-score, WC/Ht, BMI, WC, eFM, and weight.

**Figure 2:**
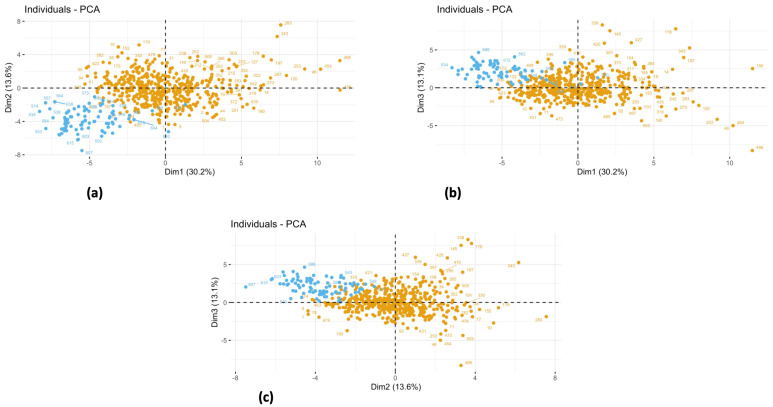
In this panels, the obesity (OB) observations (orange points) are projected in the subspaces defined by the first three principal components that explained the most variability in the dataset. The NW (light blue points) are passively projected in the subspaces. As can be observed in figure (**a**–**c**), the NW observations clustered in a region of the space where PC1 is negative.

In the next step, we analyzed the three planes defined by the PCs for the distribution of all observations in the dataset, by passively projecting the NW with the OB children. As shown in Figure 2, in the plane defined by PC1 with either of the other two dimensions, it was clear that observations related to NW children (absence of obesity) clustered in the region of the plane where PC1 is negative, in the opposite direction to where the vectors of the projections of the variables were related to the obesity point. Observations related to the group of OB children are observed much more frequently in the positive values of PC1. Overall, PC1, resulting from the principal component analysis conducted on the OB dataset and characterized by anthropometric variables, was critical in describing the differences between the group of OB children and the group of NW children, as would be expected.

Interestingly, four of the NW observations, when projected with the OB observations, were in the same region of space defined by PC1 and PC2, where the OB points were present. In fact, they showed greater values of mean insulin, HOMA-IR, fasting TG, TyG-index, and VAI than the overall NW children.

#### 3.2.2. Cluster Analysis

The hierarchical cluster analysis was conducted in concordance with the Ward method, which resulted in the definition of three clusters. We pre-specified the number of groups computed with the hierarchical algorithm in the k-means algorithm: 50 random repetitions were used. We confirmed the best number of groups found by the k-means algorithm by plotting the sum of squares of the groups in function of the number of clusters to be considered; the best trade-off was in three clusters which we called subgroup 1 (S1), subgroup 2 (S2), and subgroup 3 (S3).

**Figure 3:**
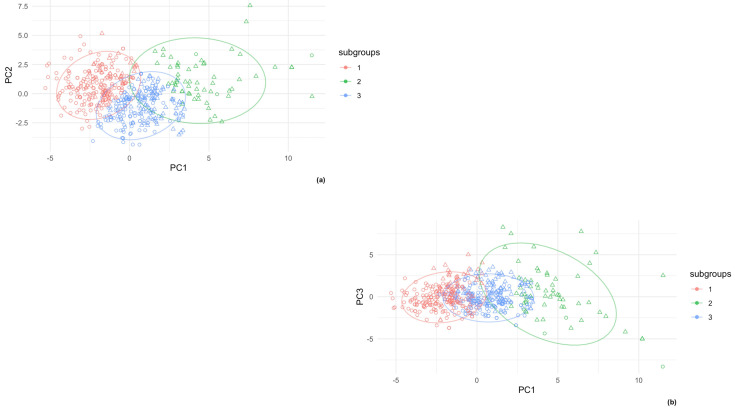
In this panel, only the obesity (OB) observations are projected in the new subspaces defined by the first three principal components obtained with multivariate analysis (PCA). Here, the observations are stratified by the inclusion of one of the three clusters obtained with the k-means algorithm (the three ellipses) and by the condition of having (triangle shape) metabolic syndrome (MS) or not (circle shape). As shown in figure (**a**) and figure (**b**), the pattern of MS mainly follows the first principal component (PC1): as the values of PC1 increase, the prevalence of MS also increases.

By projecting the observation grouped by the different clusters onto the subspaces defined by the first three principal components, it was clear that S2 and S3 were more clustered and centered, whereas S1 was more disperse, particularly in the direction of the third principal components, as shown in Figure 3. It also appears that the three subgroups differentiated in the direction of the first principal component. Moreover, if we projected the observations labeled by the condition of MS on the subspaces and compared them with the subgroups defined by the k-means algorithm, the group of MS = 1 overlapped with S1, whereas MS = 0 overlapped with S2 and S3.

The violin plots produced to explore the distribution of the anthropometric and metabolic variables in each cluster clearly showed that cluster S2 and cluster S3 had a similar profile with respect to S1, where most of the cases instead showed greater means in all the variables studied. Interestingly, we found evidence of a difference between the groups for all the anthropometric variables, except for the variables of ConI and ABSI. From Table 2, which summarizes the distributions of the three clusters, it is clear that S1 has the greatest values for the anthropometric and metabolic variables.

From examining the relative proportions of children with metabolic syndrome in the different subgroups, it appeared that some of the observations of the S1 which, if projected to the subspace defined by the PCs, should be characterized by elevated values of anthropometric and metabolic variables, still did not have MS. In addition, some children of cluster S2, the one characterized by the lowest values of PC1, were also characterized as having MS. Finally, a high proportion of the observations in S3 (intermediate) still did not have MS. We summarized the distribution of the variables for the observations of cluster S2 with MS in Table 2.

The observations of S2 with MS were characterized by lower values of eFM, waist, WHtR, and BMI z-scores, but by surprisingly higher values of TyG index, TG, VAI, HOMA-IR, and insulin. In contrast, the observations of S1 without MS were characterized by lower values of TG, insulin, HOMA-IR, and VAI, but with the highest values for eFM, WC, and WHtR. Finally, the observation of S3 without MS presented lower values for TG, insulin, HOMA-IR, and VAI, and intermediate values for eFM, waist, and WHtR.

By considering the graphs of the observations projected onto the principal components labeled for their subgroups and for the condition of MS, it seemed that the presentation of MS generally follows the first principal component, although in several cases, this pattern is not respected.

## 4. Discussion

We have presented a multivariate MVA. We noted that the metabolic risk is not only related to body weight. The variables related to IR and dyslipidemia, such as high TG levels and fat accumulation, were crucial for the early stratification of patients at risk of MS.

MS is characterized by interconnected risk factors of metabolic origin, leading to CVD and T2D and an increasing mortality risk in adult age [7,8,9,46]. One recent review reinforced the evidence that obesity (and related IR) is a consistent single risk factor for T2D; therefore, it is crucial for limiting the progression of obesity [47]. There are different dysregulated metabolic pathways (increased free fatty acid flux from adipose tissue, increased hepatic de novo lipogenesis from excessive carbohydrate consumption, and hypertriglyceridemia) that accelerate the progression from normal to impaired glucose tolerance test and diabetes [48]. Adipose tissue expansion leads to the overproduction of adipocytokines, further exacerbating metabolic dysfunction. Excesses or deficiencies of adipocytokines such as leptin or adiponectin further affect insulin sensitivity [49]. The complex interactions between glucotoxic and lipotoxic effects which ensue during IR in children with obesity can worsen IR, and consequently, exacerbate dyslipidemia and hyperglycemia due to positive feedback loops; this clearly indicates that more appropriate and complex statistical modeling should be employed [47,50].

A revision of the classification could provide a powerful tool to individualize treatment regimens and identify individuals with an increased risk of complications at diagnosis. We stratified patients into three subgroups with differing disease progression and diabetic complication risks. This new sub-stratification might represent a first step towards precision medicine in diabetes.

Due to different definitions, the MS prevalence in pediatrics remains unclear, and varies widely depending on the different definitions utilized [15]. It is reported more frequently in subjects who are overweight (11.9%) and with obesity (29.2%) [15,51,52]; however, unhealthy normal-weight patients and metabolically healthy obesity (MHO) has also been reported [28]. We noted that some of the NW observations projected with the OB observations were in the same space where the OB values were present, as defined by PC1 and PC2. On the other hand, some OB observations were far away from the mean center of the OB group and were closer to the mean center of the NW group. These data confirmed that the pathogenic mechanism of the metabolic regulation may be multifactorial, and excessive fat accumulation in tandem with a normal BMI does not protect against metabolic risks [16].

In adults, higher levels of adiponectin, lower visceral fat contents, and lower percentages of ectopic fat, such as in the muscles and liver, have been reported in MHO individuals compared with patients with metabolic complications [53,54,55]. In unhealthy normal-weight subjects, metabolic risks such as elevated glucose, insulin resistance, dyslipidemia, and hypertension are more strongly associated with high subcutaneous abdominal fat mass, visceral obesity, or fatty liver [56]. Cota et al. reported that the presence of normal-weight obesity in adolescents was associated with the accumulation of abdominal fat and an unfavorable lipid profile [16].

Our analysis supports the role of IR and dyslipidemia, such as high TG levels, and fat distribution as principal players interested in the risk stratification for MS. We noted that both the HOMA-IR and TyG Index are involved; these two indices have been proposed as simple surrogates with high sensitivity in recognizing IR and are considered as optimal predictors of metabolic and cardiovascular diseases in normoglycemic [57] and pre-diabetic patients [58,59]. Compared with other IR indicators, the TyG index mainly quantifies IR in muscle and represents a better indicator for peripheral IR, which could also be of interest in NW or underweight [60] patients in which a relatively low leg fat mass and high subcutaneous abdominal fat mass are present. Studies have shown that TyG, independent of body weight, is related to a risk of diabetes [61], hypertension [62], and non-alcoholic fatty liver disease [63], and it can predict the development of cardiovascular events [64].

We confirmed that, in addition to BMI, other anthropometric measures, such as WC and WHtR, may be useful to identify abdominal obesity. Additionally, the evaluation of new adiposity indices, particularly VAI and eFM, may be helpful in clinical practice to estimate the role of body fatness and fat distribution, when instrumentation to estimate the body composition is not available, and for the early detection of at-risk children [17,18,19,20,21,22,23,24,25,26]. In contrast, compared with the reported data in adults, blood pressure is not included in the parameters that contribute to definitions of the principal components of risk. The roles of IR and hyperinsulinemia in the pathogenesis of essential hypertension have been extensively reported [65,66]; the instauration of metabolic alterations may precede hypertension, and the time of exposure may trigger the manifestation and evolution [67,68].

In the literature, debates remain as to whether MHO represents a unique subset of people with obesity, or is simply a group which is in transition to the later development of metabolically unhealthy obesity (MUO) [69]. As reported by Blüher [70], the principal factors associated with the conversion of MHO to MUO are a decline in insulin sensitivity and an increase in fasting blood glucose [71]. In our population, the distribution of the clinical and biochemical variables stratified by the clusters clearly represented a condition in which S2 and S3 were more similar to each other with respect to S1, suggesting that a transitional phase could exist and that the IR, earlier than hyperglycemia, initiates different pathogenic pathways which increase metabolic risk and result in the full expression of the MS [72,73].

The concordant profiles of IR and triglycerides in subgroups support the notion that hyperinsulinemia could induce an increased transcription of genes for lipogenic hepatic, leading to increased TG production and influencing the severity of metabolic involvement [15]. The hypothesis that triglycerides may be an earlier dismetabolic marker could also be considered [74]. As reported elsewhere [75], hypertriglyceridemic waist phenotypes, characterized by increased TG levels and WC, can be used to predict cardiovascular risk in adult men. Despite high triglycerides levels being a modifiable cardiovascular risk factor, they contribute to oxidative damage [33] which may play a causative role in the synergistic effects of the MS components.

The MHO concept may be a model to better understand the mechanisms linking obesity with cardiometabolic diseases [76]. In pediatrics, this aspect is particularly interesting to explore, in order to define preventive measures and to adopt a tailored monitoring.

We recognize that there are some limitations to this study, starting with its retrospective cross-sectional nature. Additionally, we considered indirect indices of body fatness and fat distribution; a comparison with body compositions estimated from bio-impedance could improve the validity of our findings. Finally, we only considered anthropometric and biochemical parameters to stratify the metabolic risk; moreover, additional factors such as genetic contribution, dietary intake, physical activity level, composition, and the diversity of the microbiota could also be considered. Despite the limitations, this type of analysis is interesting to consider for the health assessments of pediatric patients with obesity.

## 5. Conclusions

Here, we considered different anthropometric measures and biochemical variables to improve the early stratification of pediatric patients at risk of MS, independently of body weight. A transitional phase from MHO to MUO may not be excluded in pediatrics.

The early detection of the interrelated variables and interventions on these modifiable risk factors is useful to protect pediatric health. Cluster analysis may help in identifying specific patient characteristics related to disease and therapeutic responses leading to a personalized medicine.

## Figures and Tables

**Table 1:** Descriptive statistics of children with obesity (OB) and the control group (NW).

Children with Obesity				Children with Normal Weight		
	Level	F	M	Level	F	M
*n*		274	254		60	59
Age (median (IQR)) (years)		10.00 (9.00, 12.00)	11.00 (9.25, 13.00)		10.00 (9.00, 11.25)	12.00 (11.00, 13.00)
Weight (median (IQR)) (kg)		58.25 (45.65, 73.50)	60.70 (49.78, 72.95)		35.55 (32.20, 39.90)	37.50 (29.75, 49.00)
Height (median (IQR)) (cm)		146.00 (136.00, 155.30)	149.00 (140.00, 157.78)		143.55 (135.70, 152.50)	146.80 (136.80, 154.30)
BMI (median (IQR)) (kg/m^2^)		27.01 (24.43, 30.25)	27.26 (25.03, 29.68)		17.50 (16.17, 18.94)	17.33 (15.77, 20.07)
WC (median (IQR)) (cm)		84.00 (79.00, 92.50)	88.20 (81.50, 94.88)		62.75 (60.00, 66.25)	61.00 (57.50, 73.00)
WC/Ht (median (IQR))		0.59 (0.55, 0.63)	0.59 (0.57, 0.63)		0.43 (0.42, 0.47)	0.44 (0.41, 0.47)
Total Cholesterol (median (IQR)) (mg/dL)		149.00 (135.00, 168.75)	154.00 (138.00, 175.75)		156.00 (130.00, 166.50)	154.00 (144.00, 174.00)
HDL Cholesterol (median (IQR)) (mg/dL)		45.00 (39.00, 52.00)	46.00 (39.00, 53.00)		56.00 (46.75, 60.00)	54.00 (49.00, 65.00)
LDL Cholesterol (median (IQR)) (mg/dL)		86.20 (72.45, 101.15)	90.20 (75.40, 106.05)		85.90 (68.65, 101.80)	87.80 (81.60, 107.80)
TG (median (IQR)) (mg/dL)		81.00 (62.00, 108.00)	72.50 (58.00, 107.00)		43.50 (34.00, 63.75)	49.00 (40.00, 57.00)
Fasting Glucose (median (IQR)) (mg/dL)		80.00 (75.00, 86.00)	84.00 (78.25, 88.00)		75.00 (67.00, 78.25)	76.00 (71.50, 82.00)
Insulin (median (IQR)) (µU/mL)		14.20 (10.43, 21.25)	13.70 (8.72, 19.70)		5.60 (4.00, 7.28)	5.00 (2.88, 8.50)
HOMA_IR (median (IQR))		2.85 (1.96, 4.24)	2.71 (1.76, 4.11)		0.99 (0.73, 1.35)	0.94 (0.51, 1.60)
Pubertal_stage (%)	1	81 (29.6)	51 (20.1)	1	14 (23.3)	12 (20.3)
	2	138 (50.4)	163 (64.2)	2	34 (56.7)	41 (69.5)
	3	55 (20.1)	40 (15.7)	3	12 (20.0)	6 (10.2)
SBP (median (IQR)) (mmHg)		110.00 (105.00, 120.00)	111.00 (105.00, 120.00)		100.00 (95.00, 106.25)	105.00 (100.00, 110.00)
DBP (median (IQR)) (mmHg)		62.00 (57.00, 70.00)	61.00 (58.00, 70.00)		60.00 (60.00, 70.00)	65.00 (60.00, 70.00)
VAI (median (IQR))		1.38 (0.99, 2.03)	1.96 (1.37, 3.01)		0.53 (0.47, 0.97)	0.86 (0.81, 1.13)
ABSI (median (IQR))		0.05 (0.04, 0.05)	0.05 (0.04, 0.05)		0.05 (0.05, 0.06)	0.05 (0.05, 0.06)
TMI (median (IQR))		18.76 (17.48, 20.26)	18.51 (17.12, 20.00)		12.04 (11.46, 13.74)	12.21 (11.36, 13.01)
ConI (median (IQR))		3.94 (3.75, 4.12)	4.05 (3.85, 4.20)		3.64 (3.54, 3.74)	3.66 (3.52, 3.79)
TyG Index (median (IQR))		8.09 (7.81, 8.40)	8.04 (7.72, 8.42)		7.36 (7.21, 7.67)	7.51 (7.19, 7.75)
eFM (median (IQR)) (kg)		24.42 (18.13, 31.23)	23.45 (19.13, 29.09)		9.75 (7.56, 11.95)	7.45 (5.35, 11.97)
BMI z-score (median (IQR))		2.74 (2.42, 3.11)	2.97 (2.57, 3.48)		0.40 (−0.42, 0.96)	0.12 (−0.77, 0.64)
MS (%)	0	197 (71.9)	136 (53.5)	0	59 (98.3)	59 (100.0)
	1	77 (28.1)	118 (46.5)	1	1 (1.7)	0 (0.0)

The continuous variables are reported as the median and IQR, whereas categorical variable are reported as frequencies and percentages.

**Table 2:** Table 2 summarizes the descriptive statistics of the OB observations stratified by the three clusters obtained with the k-means algorithm. Here, the continuous variables are reported as the median and IQR, whereas categorical variables are reported as frequencies and percentage. S1 has the greatest prevalence of MS, followed by S3 and S2. In addition, the table reports the descriptive statistics of a particular sub-cluster of S2 children which contains observations that even though are characterized by lower values of the variables positively associated with PC1, still they are classified as having MS. In fact, they show higher values in the insulin, HOMA-IR, TyG index, VAI, and fasting TG variables compared with the median values of the S2 cluster.

	Level	Children in Subgroup 1 (S1)	Children in Subgroup 2 (S2)	Children in Subgroup 3 (S3)	Children in Subgroup 2 with MS
*n*		61	230	237	44
Weight (median (IQR)) (kg)		82.50 (70.50, 95.60)	46.00 (40.15, 52.25)	69.90 (62.42, 77.00)	46.00 (42.50, 52.70)
Height (median (IQR)) (cm)		156.00 (149.50, 163.50)	136.50 (128.50, 142.00)	155.00 (150.40, 161.85)	139.00 (131.00, 145.70)
BMI (median (IQR)) (kg/m^2^)		33.50 (31.05, 36.47)	24.69 (23.45, 26.13)	28.76 (27.08, 30.55)	24.87 (23.97, 25.71)
WC (median (IQR)) (cm)		102.00 (97.00, 109.00]	80.00 (75.00, 83.88]	90.00 (86.00, 96.00]	81.50 (78.75, 83.25]
WC/Ht (median (IQR))		0.66 (0.63, 0.70)	0.59 (0.56, 0.62)	0.58 (0.54, 0.62)	0.59 (0.56, 0.63)
Total Cholesterol (median (IQR]) (mg/dL)		160.00 (139.00, 186.00]	155.50 (140.00, 177.00]	148.00 (132.00, 162.00)	152.00 (143.75, 181.00)
HDL Cholesterol (median (IQR)) (mg/dL)		39.00 (35.00, 45.00)	48.00 (41.00, 55.00)	45.00 (40.00, 51.00)	39.50 (35.00, 46.00)
LDL Cholesterol (median (IQR)) (mg/dL)		85.60 (71.40, 103.80)	91.60 (75.45, 108.00)	84.20 (73.40, 97.40)	92.20 (77.50, 109.15)
TG (median (IQR)) (mg/dL)		128.00 (102.00, 193.00)	71.00 (56.00, 94.00)	76.00 (59.00, 99.00)	124.00 (77.00, 156.25)
Fasting Glucose (median (IQR)) (mg/dL)		87.00 (80.00, 92.00)	80.00 (76.00, 85.00)	82.00 (76.00, 89.00)	82.00 (79.00, 85.00)
Insulin (median (IQR)) (µU/mL)		30.30 (22.10, 38.10)	9.95 (7.00, 14.20)	16.10 (12.20, 20.80)	13.90 (9.78, 18.10)
HOMA-IR (median (IQR))		6.30 (4.70, 7.88)	2.01 (1.40, 2.86)	3.18 (2.20, 4.31)	2.88 (2.07, 3.75)
SBP (median (IQR)) (mmHg)		123.00 (115.00, 130.00)	106.00 (100.00, 111.00)	115.00 (110.00, 122.00)	105.00 (98.00, 111.50)
DBP (median (IQR)) (mmHg)		68.00 (60.00, 74.00)	60.00 (55.00, 65.00)	65.00 (60.00, 70.00)	60.00 (53.75, 65.00)
VAI (median (IQR))		3.82 (2.20, 5.13)	1.43 (0.95, 2.04)	1.64 (1.18, 2.43)	2.97 (1.90, 4.20)
ABSI (median (IQR))		0.05 (0.04, 0.05)	0.04 (0.04, 0.05)	0.05 (0.05, 0.05)	0.05 (0.04, 0.05)
TMI (median (IQR)) (kg/m^3^)		21.56 (20.02, 24.04)	18.26 (17.12, 19.90)	18.43 (17.32, 19.80)	17.79 (16.64, 19.19)
ConI (median (IQR)) (cm)		4.16 (3.99, 4.28)	4.00 (3.82, 4.15)	3.94 (3.74, 4.11)	4.08 (3.87, 4.23)
TyG Index (median (IQR))		8.59 (8.36, 9.02)	7.97 (7.68, 8.24)	8.06 (7.78, 8.33)	8.52 (8.15, 8.77)
eFM (median (IQR)) (kg)		36.52 (32.26, 40.76)	17.84 (15.32, 20.75)	27.91 (24.50, 30.91)	17.78 (16.03, 20.17)
BMI z-score (median (IQR))		3.45 (2.98, 3.95)	2.92 (2.58, 3.55)	2.68 (2.39, 2.97)	2.83 (2.55, 3.18)
MS (%)	0	4 (6.6)	186 (80.9)	143 (60.3)	44 (100.0)
	1	57 (93.4)	44 (19.1)	94 (39.7)	

## Data Availability

The data presented in this study are available on request from the corresponding author.
